# Supplementary material for: A Proteolytic Complex Targets Multiple Cell Wall Hydrolases in Pseudomonas aeruginosa
Source: mBio. 2018 Jul 17;9(4):e00972-18. doi: 10.1128/mBio.00972-18 (PMC6050968; doi:10.1128/mBio.00972-18)
Supplement: TABLE S1 [file mbo003183912st1.pdf]

Supplemental Table 1: Proteins that co-purified in each of two LbcA•CtpA-S302A pulldowns, but were absent in each of two LbcA•CtpA pulldowns

Accession	Description	Coverage Expt. 1	# PSM Expt. 1	Coverage Expt. 2	# PSM Expt. 2	Average Coverage	Average #PSM	Gene	Confirmed CtpA substrate?
Q9I5Q4	Uncharacterized protein OS=Pseudomonas aeruginosa (strain ATCC 15692 / PAO1 / 1C / PRS 101 / LMG 12228) GN=PA0667 PE=4 SV=1 - [Q9I5Q4_PSEAE]	64.21	87	59.28	76	61.745	81.5	PA0667/mepM	YES
Q9I4E0	Uncharacterized protein OS=Pseudomonas aeruginosa (strain ATCC 15692 / PAO1 / 1C / PRS 101 / LMG 12228) GN=PA1198 PE=4 SV=1 - [Q9I4E0_PSEAE]	76.59	68	75.12	56	75.855	62	PA1198	YES
Q9I2T2	Membrane-bound lytic murein transglycosylase D OS=Pseudomonas aeruginosa (strain ATCC 15692 / PAO1 / 1C / PRS 101 / LMG 12228) GN=mltD PE=4 SV=1 - [Q9I2T2_PSEAE]	43.45	29	45.51	27	44.48	28	PA1812/mltD	
Q9X6V6	RlpA-like lipoprotein OS=Pseudomonas aeruginosa (strain ATCC 15692 / PAO1 / 1C / PRS 101 / LMG 12228) GN=rlpA PE=3 SV=1 - [RLPA_PSEAE]	71.05	24	54.39	23	62.72	23.5	PA4000/RlpA	
O87128	Chromosome/plasmid partitioning protein ParA OS=Pseudomonas aeruginosa GN=orf3 PE=4 SV=1 - [O87128_PSEAI]	40.46	19	37.02	19	38.74	19	PA1462	
Q9I0M6	Serine--tRNA ligase OS=Pseudomonas aeruginosa (strain ATCC 15692 / PAO1 / 1C / PRS 101 / LMG 12228) GN=serS PE=3 SV=1 - [SYS_PSEAE]	42.96	16	40.85	19	41.905	17.5	serS	
Q9HVL7	50S ribosomal protein L27 OS=Pseudomonas aeruginosa (strain ATCC 15692 / PAO1 / 1C / PRS 101 / LMG 12228) GN=rpmA PE=1 SV=3 - [RL27_PSEAE]	68.24	14	63.53	16	65.885	15	rpmA	
Q9HXP9	30S ribosomal protein S16 OS=Pseudomonas aeruginosa (strain ATCC 15692 / PAO1 / 1C / PRS 101 / LMG 12228) GN=rpsP PE=3 SV=1 - [RS16_PSEAE]	63.86	9	59.04	12	61.45	10.5	rpsP	
Q9HVF9	Uncharacterized protein OS=Pseudomonas aeruginosa (strain ATCC 15692 / PAO1 / 1C / PRS 101 / LMG 12228) GN=PA4632 PE=3 SV=1 - [Q9HVF9_PSEAE]	26.37	6	44.69	12	35.53	9	PA4632	
Q9I6C0	Cell division protein FtsE OS=Pseudomonas aeruginosa (strain ATCC 15692 / PAO1 / 1C / PRS 101 / LMG 12228) GN=ftsE PE=4 SV=1 - [Q9I6C0_PSEAE]	61.43	10	56.50	11	58.965	10.5	ftsE	
Q9HUH3	Uncharacterized protein OS=Pseudomonas aeruginosa (strain ATCC 15692 / PAO1 / 1C / PRS 101 / LMG 12228) GN=PA4992 PE=1 SV=1 - [Q9HUH3_PSEAE]	44.81	10	47.78	10	46.295	10	PA4992	
Q9HX31	DNA polymerase III, delta subunit OS=Pseudomonas aeruginosa (strain ATCC 15692 / PAO1 / 1C / PRS 101 / LMG 12228) GN=holA PE=4 SV=1 - [Q9HX31_PSEAE]	24.06	8	38.84	10	31.45	9	holA	
Q9HWD4	30S ribosomal protein S10 OS=Pseudomonas aeruginosa (strain ATCC 15692 / PAO1 / 1C / PRS 101 / LMG 12228) GN=rpsJ PE=3 SV=1 - [RS10_PSEAE]	53.40	7	59.22	10	56.31	8.5	rpsJ	
Q9I0T2	Probable acyl-CoA dehydrogenase OS=Pseudomonas aeruginosa (strain ATCC 15692 / PAO1 / 1C / PRS 101 / LMG 12228) GN=PA2552 PE=3 SV=1 - [Q9I0T2_PSEAE]	22.93	5	42.93	10	32.93	7.5	PA2552	
Q9HVX3	Soluble and membrane-bound lytic transglycosylase OS=Pseudomonas aeruginosa (strain ATCC 15692 / PAO1 / 1C / PRS 101 / LMG 12228) GN=mltB1 PE=4 SV=1 - [Q9HVX3_PSEAE]	23.98	7	30.25	9	27.115	8	mltB1	
Q9I5H1	Uncharacterized protein OS=Pseudomonas aeruginosa (strain ATCC 15692 / PAO1 / 1C / PRS 101 / LMG 12228) GN=PA0758 PE=4 SV=1 - [Q9I5H1_PSEAE]	29.86	6	44.96	9	37.41	7.5	PA0758	
Q9I7B8	Glycine--tRNA ligase beta subunit OS=Pseudomonas aeruginosa (strain ATCC 15692 / PAO1 / 1C / PRS 101 / LMG 12228) GN=glyS PE=3 SV=1 - [SYGB_PSEAE]	17.69	9	15.94	8	16.815	8.5	glyS	
Q9HW86	MvaT OS=Pseudomonas aeruginosa (strain ATCC 15692 / PAO1 / 1C / PRS 101 / LMG 12228) GN=mvaT PE=1 SV=1 - [Q9HW86_PSEAE]	47.58	7	40.32	8	43.95	7.5	mvaT	
Q9HWD6	50S ribosomal protein L4 OS=Pseudomonas aeruginosa (strain ATCC 15692 / PAO1 / 1C / PRS 101 / LMG 12228) GN=rplD PE=3 SV=1 - [RL4_PSEAE]	37.50	6	54.00	8	45.75	7	rplD	
Q9HWE2	50S ribosomal protein L16 OS=Pseudomonas aeruginosa (strain ATCC 15692 / PAO1 / 1C / PRS 101 / LMG 12228) GN=rplP PE=3 SV=1 - [RL16_PSEAE]	34.31	4	41.61	8	37.96	6	rplP	
Q9HWF1	50S ribosomal protein L18 OS=Pseudomonas aeruginosa (strain ATCC 15692 / PAO1 / 1C / PRS 101 / LMG 12228) GN=rplR PE=3 SV=1 - [RL18_PSEAE]	21.55	3	42.24	8	31.895	5.5	rplR	
Q9I4S6	Probable outer membrane protein OS=Pseudomonas aeruginosa (strain ATCC 15692 / PAO1 / 1C / PRS 101 / LMG 12228) GN=PA1048 PE=4 SV=1 - [Q9I4S6_PSEAE]	39.25	10	32.42	7	35.835	8.5	PA1048	
Q9I503	Uncharacterized protein OS=Pseudomonas aeruginosa (strain ATCC 15692 / PAO1 / 1C / PRS 101 / LMG 12228) GN=PA0955 PE=4 SV=1 - [Q9I503_PSEAE]	36.79	9	32.39	7	34.59	8	PA0955	
Q9I3B3	Probable chemotaxis transducer OS=Pseudomonas aeruginosa (strain ATCC 15692 / PAO1 / 1C / PRS 101 / LMG 12228) GN=PA1608 PE=4 SV=1 - [Q9I3B3_PSEAE]	25.14	8	21.44	7	23.29	7.5	PA1608	
Q9HW15	Probable short-chain dehydrogenase OS=Pseudomonas aeruginosa (strain ATCC 15692 / PAO1 / 1C / PRS 101 / LMG 12228) GN=speA PE=3 SV=1 - [Q9HW15_PSEAE]	26.98	5	41.27	7	34.125	6	speA	
Q9I5R6	2-nonaprenyl-3-methyl-6-methoxy-1,4-benzoquinol hydroxylase OS=Pseudomonas aeruginosa (strain ATCC 15692 / PAO1 / 1C / PRS 101 / LMG 12228) GN=coq7 PE=1 SV=1 - [COQ7_PSEAE]	51.16	6	41.86	6	46.51	6	PA0655	
P72176	PchC protein OS=Pseudomonas aeruginosa GN=pchC PE=4 SV=1 - [P72176_PSEAI]	20.80	4	17.60	6	19.2	5	pchC	
Q9HUW8	Uncharacterized protein OS=Pseudomonas aeruginosa (strain ATCC 15692 / PAO1 / 1C / PRS 101 / LMG 12228) GN=PA4842 PE=4 SV=1 - [Q9HUW8_PSEAE]	16.29	5	16.01	5	16.15	5	PA4842	
Q9HVF8	Probable chemotaxis transducer OS=Pseudomonas aeruginosa (strain ATCC 15692 / PAO1 / 1C / PRS 101 / LMG 12228) GN=PA4633 PE=4 SV=1 - [Q9HVF8_PSEAE]	9.27	5	11.52	5	10.395	5	PA4633	
P72158	N5-carboxyaminoimidazole ribonucleotide synthase OS=Pseudomonas aeruginosa (strain ATCC 15692 / PAO1 / 1C / PRS 101 / LMG 12228) GN=purK PE=3 SV=2 - [PURK_PSEAE]	15.00	4	18.33	5	16.665	4.5	purK	
P14532	Cytochrome c551 peroxidase OS=Pseudomonas aeruginosa (strain ATCC 15692 / PAO1 / 1C / PRS 101 / LMG 12228) GN=ccpA PE=1 SV=2 - [CCPR_PSEAE]	9.54	4	15.32	5	12.43	4.5	ccpR	
P30819	Nicotinate-nucleotide pyrophosphorylase [carboxylating] OS=Pseudomonas aeruginosa (strain ATCC 15692 / PAO1 / 1C / PRS 101 / LMG 12228) GN=nadC PE=3 SV=2 - [NADC_PSEAE]	13.12	3	19.15	5	16.135	4	nadC	
Q9HXY1	Methionine aminopeptidase OS=Pseudomonas aeruginosa (strain ATCC 15692 / PAO1 / 1C / PRS 101 / LMG 12228) GN=map PE=1 SV=1 - [Q9HXY1_PSEAE]	12.26	2	28.35	5	20.305	3.5	map	
Q9I405	Amino acid ABC transporter ATP binding protein OS=Pseudomonas aeruginosa (strain ATCC 15692 / PAO1 / 1C / PRS 101 / LMG 12228) GN=PA1339 PE=3 SV=1 - [Q9I405_PSEAE]	12.70	2	25.82	5	19.26	3.5	PA1339	
Q9I5Y1	Fructose-bisphosphate aldolase OS=Pseudomonas aeruginosa (strain ATCC 15692 / PAO1 / 1C / PRS 101 / LMG 12228) GN=fba PE=3 SV=1 - [ALF_PSEAE]	9.60	2	24.01	5	16.805	3.5	fda	
Q9HW68	Probable fumarase OS=Pseudomonas aeruginosa (strain ATCC 15692 / PAO1 / 1C / PRS 101 / LMG 12228) GN=PA4333 PE=4 SV=1 - [Q9HW68_PSEAE]	5.13	2	16.57	5	10.85	3.5	PA4333	
Q9I0I6	Methyl-accepting chemotaxis protein PA2652 OS=Pseudomonas aeruginosa (strain ATCC 15692 / PAO1 / 1C / PRS 101 / LMG 12228) GN=PA2652 PE=3 SV=1 - [MALCR_PSEAE]	2.85	2	15.33	5	9.09	3.5	PA2652	
Q9HYU8	Probable HIT family protein OS=Pseudomonas aeruginosa (strain ATCC 15692 / PAO1 / 1C / PRS 101 / LMG 12228) GN=PA3295 PE=4 SV=1 - [Q9HYU8_PSEAE]	41.38	6	17.24	4	29.31	5	PA3295	
Q9HVM1	30S ribosomal protein S20 OS=Pseudomonas aeruginosa (strain ATCC 15692 / PAO1 / 1C / PRS 101 / LMG 12228) GN=rpsT PE=1 SV=3 - [RS20_PSEAE]	34.07	6	34.07	4	34.07	5	rpsT	
Q9I2S3	Uncharacterized protein OS=Pseudomonas aeruginosa (strain ATCC 15692 / PAO1 / 1C / PRS 101 / LMG 12228) GN=fimL PE=4 SV=1 - [Q9I2S3_PSEAE]	17.08	6	11.21	4	14.145	5	fimL	
Q9HVX4	Uncharacterized protein OS=Pseudomonas aeruginosa (strain ATCC 15692 / PAO1 / 1C / PRS 101 / LMG 12228) GN=PA4441 PE=4 SV=1 - [Q9HVX4_PSEAE]	37.84	5	34.46	4	36.15	4.5	PA4441	
Q9I787	Uncharacterized protein OS=Pseudomonas aeruginosa (strain ATCC 15692 / PAO1 / 1C / PRS 101 / LMG 12228) GN=PA0045 PE=4 SV=1 - [Q9I787_PSEAE]	24.56	4	24.56	4	24.56	4	PA0045	
Q9HU24	dTDP-glucose 4,6-dehydratase OS=Pseudomonas aeruginosa (strain ATCC 15692 / PAO1 / 1C / PRS 101 / LMG 12228) GN=rmlB PE=3 SV=1 - [Q9HU24_PSEAE]	18.47	4	19.32	4	18.895	4	rmlB	
Q9I215	Probable transcriptional regulator OS=Pseudomonas aeruginosa (strain ATCC 15692 / PAO1 / 1C / PRS 101 / LMG 12228) GN=PA2100 PE=4 SV=1 - [Q9I215_PSEAE]	18.03	4	18.24	4	18.135	4	PA2100	
Q51434	Protein phosphatase CheZ OS=Pseudomonas aeruginosa (strain ATCC 15692 / PAO1 / 1C / PRS 101 / LMG 12228) GN=cheZ PE=3 SV=2 - [CHEZ_PSEAE]	17.18	4	17.18	4	17.18	4	cheZ	
Q9HW06	Probable pyrophosphohydrolase OS=Pseudomonas aeruginosa (strain ATCC 15692 / PAO1 / 1C / PRS 101 / LMG 12228) GN=PA4400 PE=4 SV=1 - [Q9HW06_PSEAE]	13.65	3	27.62	4	20.635	3.5	PA4400	
P21629	High-affinity branched-chain amino acid transport ATP-binding protein BraF OS=Pseudomonas aeruginosa (strain ATCC 15692 / PAO1 / 1C / PRS 101 / LMG 12228) GN=braF PE=3 SV=1 - [BRAF_PSEAE]	18.82	3	17.25	4	18.035	3.5	braF	
Q9HW04	Arginine biosynthesis bifunctional protein ArgJ OS=Pseudomonas aeruginosa (strain ATCC 15692 / PAO1 / 1C / PRS 101 / LMG 12228) GN=argJ PE=3 SV=1 - [ARGJ_PSEAE]	17.53	3	18.52	4	18.025	3.5	argJ	
Q9HZP7	Electron transfer flavoprotein subunit alpha OS=Pseudomonas aeruginosa (strain ATCC 15692 / PAO1 / 1C / PRS 101 / LMG 12228) GN=etfA PE=3 SV=1 - [ETFA_PSEAE]	15.86	3	20.39	4	18.125	3.5	etfA	
P07344	Tryptophan synthase alpha chain OS=Pseudomonas aeruginosa (strain ATCC 15692 / PAO1 / 1C / PRS 101 / LMG 12228) GN=trpA PE=3 SV=3 - [TRPA_PSEAE]	13.43	3	18.28	4	15.855	3.5	trpA	
Q9I3F6	Aerotaxis receptor Aer OS=Pseudomonas aeruginosa (strain ATCC 15692 / PAO1 / 1C / PRS 101 / LMG 12228) GN=aer PE=4 SV=1 - [Q9I3F6_PSEAE]	8.25	3	11.71	4	9.98	3.5	aer	
O33407	Esterase EstA OS=Pseudomonas aeruginosa (strain ATCC 15692 / PAO1 / 1C / PRS 101 / LMG 12228) GN=estA PE=1 SV=1 - [ESTA_PSEAE]	6.66	3	10.22	4	8.44	3.5	estA	
Q9I452	Probable 3-mercaptopyruvate sulfurtransferase OS=Pseudomonas aeruginosa (strain ATCC 15692 / PAO1 / 1C / PRS 101 / LMG 12228) GN=sseA PE=3 SV=1 - [THTM_PSEAE]	15.49	2	33.10	4	24.295	3	PA1292	
Q9HU67	Regulatory protein TypA OS=Pseudomonas aeruginosa (strain ATCC 15692 / PAO1 / 1C / PRS 101 / LMG 12228) GN=typA PE=4 SV=1 - [Q9HU67_PSEAE]	6.61	2	13.88	4	10.245	3	typA	
Q9I2F4	Ribokinase OS=Pseudomonas aeruginosa (strain ATCC 15692 / PAO1 / 1C / PRS 101 / LMG 12228) GN=rbsK PE=3 SV=1 - [Q9I2F4_PSEAE]	8.77	2	15.58	4	12.175	3	rbsK	
Q9HXP8	Signal recognition particle protein OS=Pseudomonas aeruginosa (strain ATCC 15692 / PAO1 / 1C / PRS 101 / LMG 12228) GN=ffh PE=3 SV=1 - [Q9HXP8_PSEAE]	2.84	2	8.53	4	5.685	3	ffh	
Q9HU35	Uncharacterized protein OS=Pseudomonas aeruginosa (strain ATCC 15692 / PAO1 / 1C / PRS 101 / LMG 12228) GN=PA5149 PE=4 SV=1 - [Q9HU35_PSEAE]	31.94	8	11.39	3	21.665	5.5	PA5149	
Q9I530	D-lactate dehydrogenase (Fermentative) OS=Pseudomonas aeruginosa (strain ATCC 15692 / PAO1 / 1C / PRS 101 / LMG 12228) GN=ldhA PE=3 SV=1 - [Q9I530_PSEAE]	31.00	7	19.15	3	25.075	5	ldhA	
P13982	Carbamate kinase OS=Pseudomonas aeruginosa (strain ATCC 15692 / PAO1 / 1C / PRS 101 / LMG 12228) GN=arcC PE=3 SV=1 - [ARCC_PSEAE]	17.10	6	9.68	3	13.39	4.5	arcC	
Q9HYD4	Uncharacterized protein OS=Pseudomonas aeruginosa (strain ATCC 15692 / PAO1 / 1C / PRS 101 / LMG 12228) GN=PA3472 PE=4 SV=1 - [Q9HYD4_PSEAE]	33.33	4	27.27	3	30.3	3.5	PA3472	
Q9I381	Uncharacterized protein OS=Pseudomonas aeruginosa (strain ATCC 15692 / PAO1 / 1C / PRS 101 / LMG 12228) GN=PA1644 PE=4 SV=1 - [Q9I381_PSEAE]	27.23	3	11.88	3	19.555	3	PA1644	
Q9I5E2	2-methylisocitrate lyase OS=Pseudomonas aeruginosa (strain ATCC 15692 / PAO1 / 1C / PRS 101 / LMG 12228) GN=prpB PE=3 SV=1 - [Q9I5E2_PSEAE]	18.46	3	18.46	3	18.46	3	prpB	
Q9HXK8	Uncharacterized protein OS=Pseudomonas aeruginosa (strain ATCC 15692 / PAO1 / 1C / PRS 101 / LMG 12228) GN=PA3787 PE=1 SV=1 - [Q9HXK8_PSEAE]	18.09	3	17.73	3	17.91	3	PA3787	
O82852	Uridylate kinase OS=Pseudomonas aeruginosa (strain ATCC 15692 / PAO1 / 1C / PRS 101 / LMG 12228) GN=pyrH PE=3 SV=2 - [PYRH_PSEAE]	21.63	3	20.00	3	20.815	3	pyrH	
Q57003	PilV protein OS=Pseudomonas aeruginosa GN=pilV PE=4 SV=1 - [Q57003_PSEAI]	18.38	3	18.38	3	18.38	3	pilV	
Q9HWC6	50S ribosomal protein L1 OS=Pseudomonas aeruginosa (strain ATCC 15692 / PAO1 / 1C / PRS 101 / LMG 12228) GN=rplA PE=3 SV=1 - [RL1_PSEAE]	18.18	3	18.18	3	18.18	3	rplA	
Q9HW29	Uncharacterized protein OS=Pseudomonas aeruginosa (strain ATCC 15692 / PAO1 / 1C / PRS 101 / LMG 12228) GN=PA4373 PE=4 SV=1 - [Q9HW29_PSEAE]	10.99	3	12.91	3	11.95	3	PA4373	
Q9I5W0	DNA primase OS=Pseudomonas aeruginosa (strain ATCC 15692 / PAO1 / 1C / PRS 101 / LMG 12228) GN=dnaG PE=3 SV=1 - [DNAG_PSEAE]	8.43	3	9.04	3	8.735	3	dnaG	
Q9I783	Uncharacterized protein PA0049 OS=Pseudomonas aeruginosa (strain ATCC 15692 / PAO1 / 1C / PRS 101 / LMG 12228) GN=PA0049 PE=1 SV=1 - [Y049_PSEAE]	9.04	3	9.04	3	9.04	3	PA0049	
Q9HX92	Probable transcriptional regulator OS=Pseudomonas aeruginosa (strain ATCC 15692 / PAO1 / 1C / PRS 101 / LMG 12228) GN=PA3921 PE=4 SV=1 - [Q9HX92_PSEAE]	7.06	3	7.17	3	7.115	3	PA3921	
Q00514	Type II secretion system protein G OS=Pseudomonas aeruginosa (strain ATCC 15692 / PAO1 / 1C / PRS 101 / LMG 12228) GN=xcpT PE=1 SV=1 - [GSPG_PSEAE]	39.44	2	38.73	3	39.085	2.5	xcpT	
Q9I5V8	30S ribosomal protein S21 OS=Pseudomonas aeruginosa (strain ATCC 15692 / PAO1 / 1C / PRS 101 / LMG 12228) GN=rpsU PE=1 SV=3 - [RS21_PSEAE]	35.21	2	35.21	3	35.21	2.5	rpsU	
Q9HT81	Probable GTP-binding protein EngB OS=Pseudomonas aeruginosa (strain ATCC 15692 / PAO1 / 1C / PRS 101 / LMG 12228) GN=engB PE=3 SV=1 - [ENGB_PSEAE]	15.35	2	26.05	3	20.7	2.5	PA5492	
Q9HWF4	50S ribosomal protein L15 OS=Pseudomonas aeruginosa (strain ATCC 15692 / PAO1 / 1C / PRS 101 / LMG 12228) GN=rplO PE=3 SV=1 - [RL15_PSEAE]	18.06	2	22.92	3	20.49	2.5	rplO	
Q9HZP6	Electron transfer flavoprotein subunit beta OS=Pseudomonas aeruginosa (strain ATCC 15692 / PAO1 / 1C / PRS 101 / LMG 12228) GN=etfB PE=3 SV=1 - [ETFB_PSEAE]	12.85	2	21.29	3	17.07	2.5	etfB	
Q9I031	Probable ATP-binding component of ABC transporter OS=Pseudomonas aeruginosa (strain ATCC 15692 / PAO1 / 1C / PRS 101 / LMG 12228) GN=PA2812 PE=3 SV=1 - [Q9I031_PSEAE]	13.87	2	20.97	3	17.42	2.5	PA2812	
Q9HUL7	N-acetylmuramoyl-L-alanine amidase OS=Pseudomonas aeruginosa (strain ATCC 15692 / PAO1 / 1C / PRS 101 / LMG 12228) GN=amiB PE=4 SV=1 - [Q9HUL7_PSEAE]	9.68	2	17.68	3	13.68	2.5	amiB	
Q9LCT6	D-alanine--D-alanine ligase B OS=Pseudomonas aeruginosa (strain ATCC 15692 / PAO1 / 1C / PRS 101 / LMG 12228) GN=ddlB PE=3 SV=1 - [DDLB_PSEAE]	10.97	2	16.61	3	13.79	2.5	ddlB	
Q9HTJ6	Probable two-component response regulator OS=Pseudomonas aeruginosa (strain ATCC 15692 / PAO1 / 1C / PRS 101 / LMG 12228) GN=PA5364 PE=4 SV=1 - [Q9HTJ6_PSEAE]	12.33	2	12.33	3	12.33	2.5	PA5364	
Q9I2R6	Uncharacterized protein OS=Pseudomonas aeruginosa (strain ATCC 15692 / PAO1 / 1C / PRS 101 / LMG 12228) GN=PA1829 PE=4 SV=1 - [Q9I2R6_PSEAE]	6.46	2	10.39	3	8.425	2.5	PA1829	

Accession	Description	Coverage Expt. 1	# PSM Expt. 1	Coverage Expt. 2	# PSM Expt. 2	Average Coverage	Average #PSM	Gene	Confirmed CtpA substrate?
Q9HXF4	Uncharacterized protein OS=Pseudomonas aeruginosa (strain ATCC 15692 / PAO1 / 1C / PRS 101 / LMG 12228) GN=PA3852 PE=4 SV=1 - [Q9HXF4_PSEAE]	9.97	2	9.97	3	9.97	2.5	PA3852	
Q9I4S9	Uncharacterized protein OS=Pseudomonas aeruginosa (strain ATCC 15692 / PAO1 / 1C / PRS 101 / LMG 12228) GN=PA1045 PE=4 SV=1 - [Q9I4S9_PSEAE]	4.20	2	6.02	3	5.11	2.5	PA1045	
Q9LCT4	Membrane protein OS=Pseudomonas aeruginosa GN=spoIIQ PE=4 SV=1 - [Q9LCT4_PSEAI]	17.26	4	17.26	2	17.26	3	PA4404	**YES**
Q51390	Glycerol kinase 2 OS=Pseudomonas aeruginosa (strain ATCC 15692 / PAO1 / 1C / PRS 101 / LMG 12228) GN=glpK2 PE=3 SV=2 - [GLPK2_PSEAE]	10.69	4	7.52	2	9.105	3	glpK	
Q9HV35	S-adenosylmethionine decarboxylase proenzyme OS=Pseudomonas aeruginosa (strain ATCC 15692 / PAO1 / 1C / PRS 101 / LMG 12228) GN=speH PE=3 SV=1 - [SPEH_PSEAE]	21.88	3	11.88	2	16.88	2.5	PA4773	
Q9I6F6	Uncharacterized protein OS=Pseudomonas aeruginosa (strain ATCC 15692 / PAO1 / 1C / PRS 101 / LMG 12228) GN=PA0335 PE=4 SV=1 - [Q9I6F6_PSEAE]	21.66	3	16.13	2	18.895	2.5	PA0335	
Q9HZ65	Methylthioribose-1-phosphate isomerase OS=Pseudomonas aeruginosa (strain ATCC 15692 / PAO1 / 1C / PRS 101 / LMG 12228) GN=mtnA PE=3 SV=1 - [MTNA_PSEAE]	17.60	3	14.80	2	16.2	2.5	PA3169	
Q51508	Salicylate biosynthesis isochorismate synthase OS=Pseudomonas aeruginosa (strain ATCC 15692 / PAO1 / 1C / PRS 101 / LMG 12228) GN=pchA PE=1 SV=1 - [PCHA_PSEAE]	9.87	3	6.30	2	8.085	2.5	pchA	
P42805	4-diphosphocytidyl-2-C-methyl-D-erythritol kinase OS=Pseudomonas aeruginosa (strain ATCC 15692 / PAO1 / 1C / PRS 101 / LMG 12228) GN=ispE PE=3 SV=2 - [ISPE_PSEAE]	12.41	3	6.38	2	9.395	2.5	ipk	
Q51426	Holliday junction ATP-dependent DNA helicase RuvB OS=Pseudomonas aeruginosa (strain ATCC 15692 / PAO1 / 1C / PRS 101 / LMG 12228) GN=ruvB PE=3 SV=2 - [RUVB_PSEAE]	8.81	3	6.53	2	7.67	2.5	ruvB	
Q9I000	Probable aldolase OS=Pseudomonas aeruginosa (strain ATCC 15692 / PAO1 / 1C / PRS 101 / LMG 12228) GN=PA2843 PE=4 SV=1 - [Q9I000_PSEAE]	8.04	3	6.70	2	7.37	2.5	PA2843	
Q9HXE7	Carboxylesterase OS=Pseudomonas aeruginosa (strain ATCC 15692 / PAO1 / 1C / PRS 101 / LMG 12228) GN=PA3859 PE=1 SV=1 - [Q9HXE7_PSEAE]	22.33	2	21.40	2	21.865	2	PA3859	
P34003	Shikimate kinase OS=Pseudomonas aeruginosa (strain ATCC 15692 / PAO1 / 1C / PRS 101 / LMG 12228) GN=aroK PE=3 SV=2 - [AROK_PSEAE]	17.44	2	17.44	2	17.44	2	aroK	
Q9I022	Probable glutathione S-transferase OS=Pseudomonas aeruginosa (strain ATCC 15692 / PAO1 / 1C / PRS 101 / LMG 12228) GN=PA2821 PE=3 SV=1 - [Q9I022_PSEAE]	14.55	2	14.55	2	14.55	2	PA2821	
Q9I0L1	High frequency lysogenization protein HflD homolog OS=Pseudomonas aeruginosa (strain ATCC 15692 / PAO1 / 1C / PRS 101 / LMG 12228) GN=hflD PE=3 SV=1 - [HFLD_PSEAE]	14.08	2	14.08	2	14.08	2	PA2627	
Q9HU43	1-(5-phosphoribosyl)-5-[(5-phosphoribosylamino)methylideneamino] imidazole-4-carboxamide isomerase OS=Pseudomonas aeruginosa (strain ATCC 15692 / PAO1 / 1C / PRS 101 / LMG 12228) GN=hisA PE=3 SV=1 - [HIS4_PSEAE]	11.02	2	11.02	2	11.02	2	hisA	
Q9HU12	Probable hydrolase OS=Pseudomonas aeruginosa (strain ATCC 15692 / PAO1 / 1C / PRS 101 / LMG 12228) GN=PA5177 PE=4 SV=1 - [Q9HU12_PSEAE]	10.41	2	10.41	2	10.41	2	PA5177	
Q9I0T9	Exodeoxyribonuclease III OS=Pseudomonas aeruginosa (strain ATCC 15692 / PAO1 / 1C / PRS 101 / LMG 12228) GN=xthA PE=4 SV=1 - [Q9I0T9_PSEAE]	10.37	2	7.04	2	8.705	2	xthA	
Q9HVD6	Uncharacterized protein OS=Pseudomonas aeruginosa (strain ATCC 15692 / PAO1 / 1C / PRS 101 / LMG 12228) GN=PA4656 PE=4 SV=1 - [Q9HVD6_PSEAE]	10.16	2	10.16	2	10.16	2	PA4656	
Q9HXG6	Probable ATP-binding component of ABC transporter OS=Pseudomonas aeruginosa (strain ATCC 15692 / PAO1 / 1C / PRS 101 / LMG 12228) GN=PA3838 PE=3 SV=1 - [Q9HXG6_PSEAE]	9.85	2	9.85	2	9.85	2	PA3838	
Q9I4P4	Peptidoglycan hydrolase FlgJ OS=Pseudomonas aeruginosa (strain ATCC 15692 / PAO1 / 1C / PRS 101 / LMG 12228) GN=flgJ PE=3 SV=1 - [FLGJ_PSEAE]	6.50	2	6.50	2	6.5	2	flgJ	
