# Supplementary material for: A Proteolytic Complex Targets Multiple Cell Wall Hydrolases in Pseudomonas aeruginosa
Source: mBio. 2018 Jul 17;9(4):e00972-18. doi: 10.1128/mBio.00972-18 (PMC6050968; doi:10.1128/mBio.00972-18)
Supplement: FIG S1 [file mbo003183912sf1.pdf]

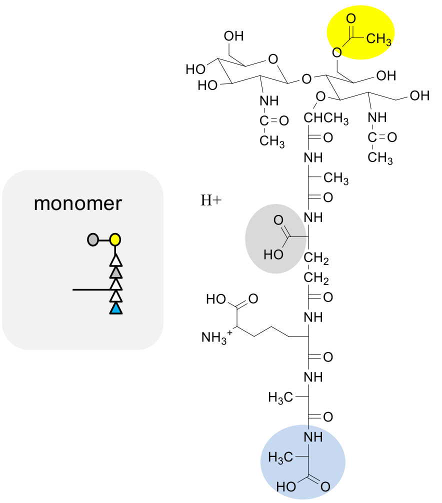

Chemical Formula: $C_{42}H_{72}N_8O_{23}^{2+}$
Exact Mass: 1056.47
Molecular Weight: 1057.07
m/z: 528.23 (100.0%), 528.74 (45.4%), 529.24 (10.1%), 529.24 (4.7%), 528.73 (3.0%), 529.74 (2.1%), 529.74 (1.5%), 529.24 (1.3%)

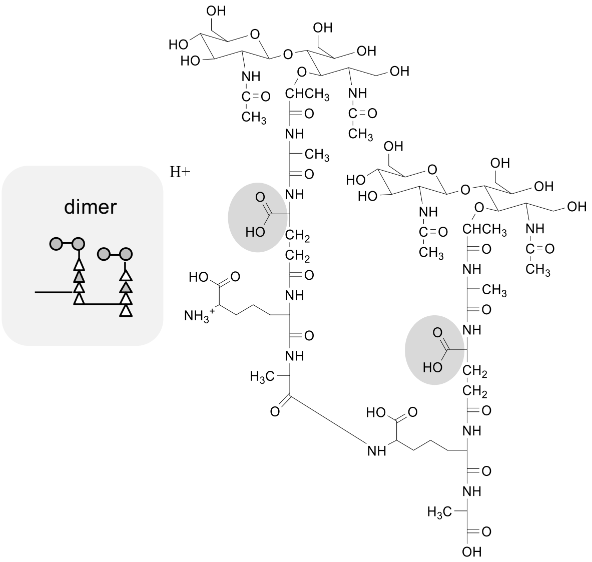

Chemical Formula: $C_{74}H_{126}N_{14}O_{41}^{2+}$
Exact Mass: 1866.82
Molecular Weight: 1867.88
m/z: 933.41 (100.0%), 933.91 (80.0%), 934.41 (31.6%), 934.41 (8.4%), 934.91 (7.4%), 934.91 (6.7%), 933.91 (5.2%), 934.41 (3.8%), 935.42 (2.7%), 934.91 (1.6%), 935.42 (1.6%), 933.91 (1.6%), 933.91 (1.4%), 934.41 (1.3%), 934.41 (1.2%)

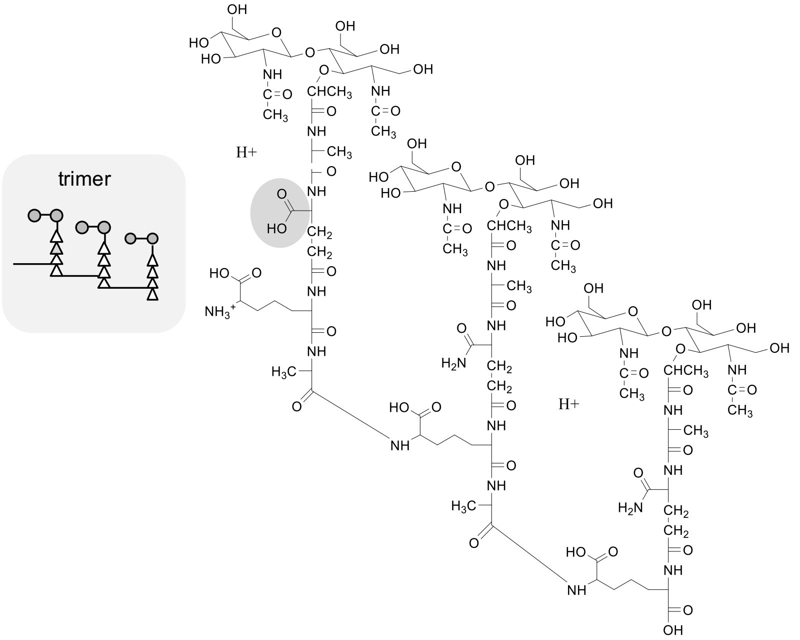

Chemical Formula: $C_{108}H_{185}N_{22}O_{58}^{3+}$
Exact Mass: 2718.22
Molecular Weight: 2719.76
m/z: 906.41 (100.0%), 906.07 (85.6%), 906.74 (36.6%), 906.74 (21.3%), 907.08 (19.8%), 907.08 (8.6%), 906.74 (8.1%), 906.74 (7.4%), 906.41 (7.0%), 907.41 (6.1%), 907.07 (4.7%), 907.41 (4.4%), 907.08 (3.3%), 906.74 (2.8%), 907.41 (2.5%), 907.74 (2.4%), 907.08 (2.3%), 906.74 (2.1%), 906.41 (1.9%), 906.41 (1.8%), 906.74 (1.6%), 907.75 (1.4%), 907.08 (1.3%)

Supplementary Figure S1. Chemical structure of peptidoglycan monomer, dimer, and trimer corresponding to the mass spectra shown in Figure 6.
